# An Adaptive *Chlamydia trachomatis*-Specific IFN-γ-Producing CD4^+^ T Cell Response Is Associated With Protection Against Chlamydia Reinfection in Women

**DOI:** 10.3389/fimmu.2018.01981

**Published:** 2018-09-07

**Authors:** Rakesh K. Bakshi, Kanupriya Gupta, Stephen J. Jordan, Xiaofei Chi, Shelly Y. Lensing, Christen G. Press, William M. Geisler

**Affiliations:** ^1^Division of Infectious Diseases, Department of Medicine, University of Alabama at Birmingham, Birmingham, AL, United States; ^2^Division of Infectious Diseases, Department of Medicine, Indiana University School of Medicine, Indianapolis, IN, United States; ^3^Department of Biostatistics, University of Arkansas for Medical Sciences, Little Rock, AR, United States

**Keywords:** *Chlamydia trachomatis*, CD4+IFN-γ responses, reinfection, protection, T cell responses

## Abstract

**Background:** Adaptive immune responses that mediate protection against *Chlamydia trachomatis* (CT) remain poorly defined in humans. Animal chlamydia models have demonstrated that CD4^+^ Th1 cytokine responses mediate protective immunity against reinfection. To better understand protective immunity to CT in humans, we investigated whether select CT-specific CD4^+^ Th1 and CD8^+^ T cell cytokine responses were associated with protection against CT reinfection in women.

**Methods:** Peripheral blood mononuclear cells were collected from 135 CT-infected women at treatment and follow-up visits and stimulated with CT antigens. CD4^+^ and CD8^+^ T-cells expressing IFN-γ, TNF-α, and/or IL-2 were assessed using intracellular cytokine staining and cytokine responses were compared between visits and between women with vs. without CT reinfection at follow-up.

**Results:** A CD4^+^TNF-α response was detected in the majority (77%) of study participants at the treatment visit, but a lower proportion had this response at follow-up (62%). CD4^+^ IFN-γ and CD4^+^ IL-2 responses occurred less frequently at the treatment visit (32 and 18%, respectively), but increased at follow-up (51 and 41%, respectively). CD8^+^ IFN-γ and CD8^+^ TNF-α responses were detected more often at follow-up (59% for both responses) compared to the treatment visit (30% for both responses). At follow-up, a CD4^+^IFN-γ response was detected more often in women without vs. with reinfection (60 vs. 33%, *P* = 0.005).

**Conclusions:** Our findings suggest that a CT-specific CD4^+^ IFN-γ response is associated with protective immunity against CT reinfection and is thus an important component of adaptive immunity to CT in women.

## Introduction

*Chlamydia trachomatis* (CT), an obligate intracellular bacterium that infects human mucosal columnar epithelial cells, causes the most common bacterial sexually transmitted infection (STI) globally, with more than 131 million new CT infections annually (1). Despite prevention and control measures, CT rates continue to rise in the U.S. and are currently at the highest reported rates (2). CT infection is often asymptomatic and, if it remains untreated, can ascend to the upper genital tract and cause inflammation, which can lead to long-term complications such as infertility, chronic pelvic pain, and ectopic pregnancy (3, 4). While early antibiotic treatment can clear CT infection and reduce the risk for CT-associated complications (5), it can also potentially hinder the development of protective immunity (6), thereby leaving individuals vulnerable to subsequent reinfections, which occur in up to 20% of women after treatment of CT infection (7). Arrested immunity with early treatment has been demonstrated in the murine chlamydia model (8), and a few studies in humans support the arrested immunity hypothesis (6, 9–11). One of these studies demonstrated that women who cleared CT infection before treatment (i.e., spontaneous resolution) were 4-fold less likely to have reinfection at follow-up compared with those with persisting CT infection at the time of treatment (10), suggesting early treatment hindered adaptive immunity in some individuals thereby increasing their reinfection risk. An increase in reinfection rates is likely a major contributing factor in the continued rise in CT infection rates (6). The notion that CT screening programs with early treatment efforts are contributing to the rise in CT infection highlights the need for a preventative CT vaccine. However, CT vaccine efforts have been hampered in part by the lack of identification of the key immune correlates of protection against CT in humans.

Most of our knowledge of the immune mechanisms that protect against chlamydial infection has come from animal models, especially murine models of *C. muridarum* or CT infection. Murine models have demonstrated that antigen-specific CD4^+^ T helper type 1 (Th1) responses are crucial for eradication of primary chlamydial infection and important in inducing a protective memory immune response, while neither CD8^+^ cells nor antibody alone are necessary for clearing primary infection (12–15). Whether humans utilize the same immune mechanisms as animals to eradicate CT infection and develop protective immunity against reinfection remains to be elucidated. In a recent small study of adolescent females looking only at IFN-γ responses, Russell et al. reported women who remained uninfected over follow-up displayed a greater frequency of IFN-γ-producing CD4^+^ T cells but not CD8^+^ T cells compared to women who had incident infection (16). In another study, which evaluated female sex workers, CT-specific IFN-γ and IL-13 responses were associated with a decreased incidence of CT infection (17). Thus, there are sparse human studies evaluating CT-specific immune responses and they have been limited by small sample size and/or lack of consensus with respect to what cytokine is associated with protective immunity. Also, the previous studies have not systematically analyzed the changes in CT-specific T cell responses (both CD4^+^ and CD8^+^) over time, nor evaluated cell-specific immune correlates of protection against CT reinfection in humans using a sensitive approach like intracellular cytokine staining (ICS).

We previously used ICS to demonstrate that the majority of CT-infected women mounted a CD4^+^TNF-α response before treatment, and CD4^+^IFN-γ T cell responses were infrequent and, in general, of low magnitude at that time point; we also found heterogeneous T cell responses based on different CT antigens used in the stimulation (18). Our current study builds on this initial work by investigating changes in CT-specific T cell responses from the time of treatment to follow-up to better understand the adaptive immune response to CT infection and to evaluate whether specific immune responses are associated with lower rates of CT reinfection at follow-up. Thus, we sought to identify potential immune correlates of protection against CT reinfection.

## Methods

### Study population and procedures

This study evaluated 135 women from the study cohort described in our previous study (18). Briefly, females ≥16 years of age presenting to the Jefferson County Department of Health (JCDH) STD clinic for treatment of a recent positive screening CT NAAT (Hologic Aptima Combo 2 [AC2]; Hologic, Inc., Marlborough, MA) were invited to participate in a study on immune responses to CT infection. Exclusion criteria were pregnancy, prior hysterectomy, co-infection with HIV, syphilis, or gonorrhea (tested at screening), immunosuppressed, or had received antibiotics with anti-CT activity in the prior 30 days. Patients interested in the study provided written consent and were enrolled.

At enrollment, participants were interviewed for sociodemographic information and the following specimens were collected: a vaginal swab (for a wet mount), a cervical swab (for CT, *Neisseria gonorrhoeae*, and *Trichomonas vaginalis* testing by AC2), and blood (for isolation of peripheral blood mononuclear cells [PBMCs]). All participants received azithromycin 1g orally (directly observed) and were advised to refer all sexual partners for treatment if not already treated. All participants were scheduled for 3- and 6-month follow-up visits, at which time they had an interview and a cervical swab (for CT testing) and blood (for PBMCs) were collected again. CT NAAT (AC2) was performed at the follow-up visits to screen for CT reinfection; a test of cure had not been done after the treatment visit as it is not CDC recommended in non-pregnant women after CT treatment (19) and also azithromycin is highly efficacious for urogenital CT infection (>97% cure rate) (20). For the current study, we evaluated all women found to have CT reinfection to date (*n* = 45) and a matched group of women without reinfection (*n* = 90) that were matched in a 1:2 ratio by age, race, and follow-up duration (i.e., we evaluated the follow-up visit [3- or 6-month visit] in which a reinfected participant had their reinfection detected and used the same follow-up visit for the matched participant without reinfection).

### PBMC isolation

Ficoll gradient centrifugation was used to isolate PBMCs from blood at the University of Alabama at Birmingham (UAB) Center for Clinical and Translational Sciences Specimen Processing and Analytical Nexus (18, 21). Upon isolation, cells were counted and examined for viability. PBMCs were frozen in 1mL aliquots in 90% FBS+10% DMSO and cryopreserved in liquid nitrogen until thawed for immunological studies.

### Intracellular cytokine staining (ICS)

PBMCs were stimulated with CT antigens and analyzed for cytokines as previously reported (18, 21). Briefly, 2.5 × 10^5^ cells were incubated for 2 h at 37°C with 5% CO_2_ in RPMI-10 media (RPMI 1640 medium containing 10% human AB serum, penicillin/streptomycin [50 U/mL], HEPES [25 mM] and L-glutamine [2mM]) in the presence of antibodies reactive to co-stimulatory molecules CD28 and CD49d (BD Biosciences, San Diego, CA) and antigen, followed by a 5-h incubation in the presence of Brefeldin A and Monensin (both from BD Biosciences, San Diego, CA). Antigens used were: recombinant CT Pgp3 (5 μg/ml; Biorbyt, San Francisco, CA), pooled CT major outer membrane protein (MOMP) peptides (5 μg/ml; VS1 75-92, VS2 132-151, VS2 145-163, VS4 300-318, VS4 308-324; UAB Peptide Core, Birmingham, AL), and formalin-fixed CT elementary bodies (EBs; 4 μg/ml) (pooled CT EBs from serotypes D, F and J; obtained from Dr. Richard Morrison from the University of Arkansas for Medical Sciences, Little Rock, AR); *Staphylococcus* enterotoxin B (SEB, Toxin Technologies, Carasota, FL) was used as the positive control. RPMI-10 supplemented with anti-CD28/CD49d antibodies, but without antigen, was used to determine background T-cell responses. Cells were subsequently labeled with LIVE/DEAD fluorescent reactive dye (Life Technologies, Eugene, OR), stained with surface antibodies reactive against surface molecules CD3 (conjugated with Pacific Blue; BD biosciences, San Diego, CA), CD4 (conjugated with Qdot 655), and CD8 (conjugated with Qdot 605; both from Invitrogen, Carlsbad, CA), fixed and permeabilized (Cytofix/Cytoperm, BD Biosciences, San Diego, CA), and intracellular cytokines stained with antibodies reactive against IFN-γ (conjugated with Alexa 700), TNF-α (conjugated with PE-Cy7) and IL-2 (conjugated with PE; all from BD Biosciences, San Diego, CA). Approximately 100,000 events were acquired on a LSRII (BD Immunocytometry Systems, San Diego, CA) and data were analyzed using FlowJo software (v9.8.5, TreeStar, Ashland, OR). All responses are reported after subtracting the background responses (media with antibodies against co-stimulatory receptors was the control). A positive cytokine response was defined as a response with >0.05% cytokine-producing cells and 2-fold higher than the background (media with co-stimulatory antibodies).

### Statistical analysis

Given the design, analyses took into account the 2:1 matching of women without reinfection to those with reinfection, resulting in clusters of size 3. For paired qualitative cytokine response data, the change in the proportion of women with cytokine responses from the treatment to follow-up visits were evaluated using an extension of McNemar's chi-square test that addresses clustering (22). For unpaired qualitative cytokine response data, differences in the proportion of women with a positive cytokine response between reinfection and no reinfection groups at follow-up were evaluated in a logistic model using a generalized linear mixed models (GLMMs) approach with visit as a main effect and matched group as a random effect, which accounted for clustering of data. For quantitative cytokine response data, cytokine responses were log-transformed given the skewed distribution. A mixed model approach was used that accounted for clustering with random matched group and/or participant effects as appropriate. Association of participant characteristics with reinfection status were examined one at a time using a logistic GLMM with a given characteristic as a fixed main effect and matched group as a random effect. *P*-values <0.05 were considered statistically significant. Analyses were performed with R software (i.e., Obuchowski's test, version 3.3.3) and SAS software (version 9.3; SAS Institute, Cary, NC).

### Study approval and participant consent

This study was approved by the UAB Institutional Review Board and JCDH. All study participants provided written consent before they were enrolled in the study.

## Results

### Participant characteristics

Of the 135 women evaluated (45 with reinfection at follow-up and 90 without reinfection), the median age at enrollment was 22 years (range 16–32) and the majority were African-American and non-Hispanic (Table 1). Seventy-eight (57.8%) women had prior chlamydia (by self-report and/or review of medical records) and there was a trend towards a higher reinfection frequency in women without prior CT (*P* = 0.070). Sixty-six (48.9%) women were diagnosed with other co-infections (bacterial vaginosis, trichomoniasis, or vaginal candidiasis) and these co-infections did not significantly differ between women with and without CT reinfection at follow-up.

**Table 1:** Baseline participant characteristics, stratified by chlamydia reinfection status at follow-up.

**Characteristics**	**Total (*n* = 135)**	**Reinfection (*n* = 45)**	**No reinfection (*n* = 90)**
Age (yr), median (range)	22 (16–32)	22 (16–32)	22 (16–29)
**RACE**, ***n*** **(%)**			
African-American	133 (98.5%)	44 (97.7%)	89 (98.9%)
Caucasian	2 (1.5%)	1 (2.3%)	1 (1.1%)
**ETHNICITY**, ***n*** **(%)**			
Non-Hispanic	134 (99.3%)	44 (97.7%)	90 (100%)
Hispanic	1 (0.7%)	1 (2.3%)	0 (0%)
Use of hormonal contraception, *n* (%)	61 (45.2%)	25 (55.6%)	36 (40%)
Prior chlamydia, *n* (%)	78 (57.8%)	21 (46.7%)	57 (63.3%)
Cervicitis, *n* (%)	20 (14.8%)	9 (20%)	11 (12.2%)
**CO-INFECTION**, ***n*** **(%)**			
Bacterial Vaginosis	41 (30.4%)	14 (31.1%)	27 (30%)
Trichomoniasis	8 (5.9%)	5 (11.1%)	3 (3.3%)
Candidiasis	17 (12.6%)	2 (4.4%)	15 (16.7%)

*There was trend towards lower reinfection frequency in those with prior CT (P = 0.070), otherwise other characteristics were not associated with reinfection status (P > 0.1)*.

As described in the methods, non-reinfected participants were matched in a 2:1 ratio to reinfected participants by follow-up visit. For the 135 women, we evaluated data at 93 3-month follow-up visits (31 visits from reinfected participants and 62 from those without infection) and 42 6-month visits (14 from reinfected participants and 28 from nonreinfected subjects). There was no significant difference in frequency of reporting being sexually active since treatment in women with vs. without reinfection at follow-up (43 [95.5%] vs. 81 [90%]; *P* = 0.282), nor was there a difference in the median number of sexual partners at follow-up (for both groups, the median number was 1 [range 1–5]). Reinfected women more often reported having unprotected sex since treatment compared with women without reinfection (39 [86.7%] vs. 55 [61.1%]; *P* = 0.005). There was no significant difference in frequency of reporting that the partner received CT treatment in women with vs. without reinfection (31 [68.9%] vs. 62 [68.9%], *P* = 0.927), nor was there a significant difference in frequency of a reported new partner since treatment (18 [40%] vs. 27 [30%]; *P* = 0.214).

### The Th1 cytokine hierarchy transitioned from TNF-α-producing CD4^+^ T Cells to IFN-γ-producing CD4^+^ T cells after CT-infected women were treated

**Figure 1:**
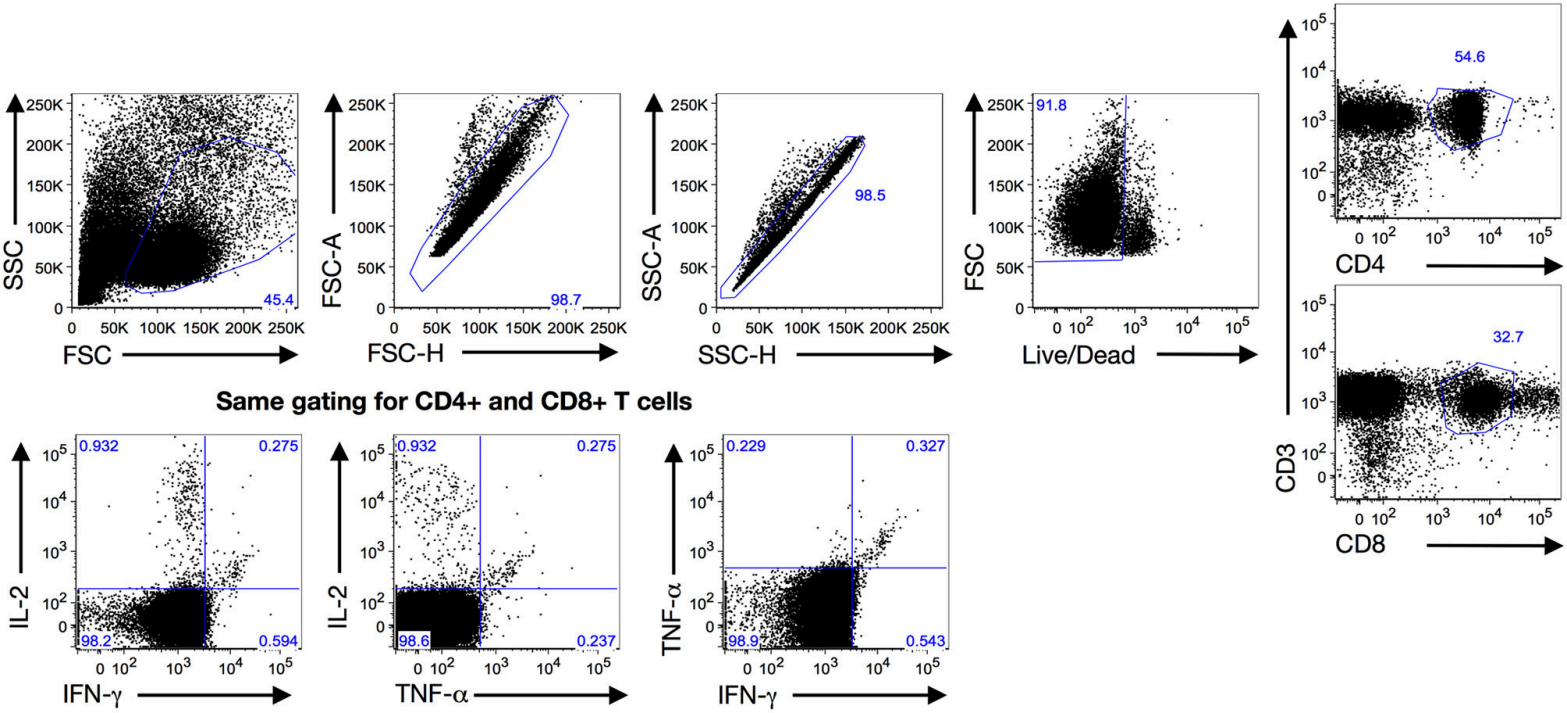
**Gating strategy:** Gating strategy used to analyze the quantitative and qualitative T cell cytokine responses at both treatment and follow-up visits after a 7-h PBMC stimulation with *Chlamydia trachomatis* (CT) antigens (Pgp3, MOMP and EB) by intracellular cytokine staining with flow cytometry. Expanded lymphocytes were first selected based upon forward (FSC) and side scatters (SSC) before gating for a singlet population of live cells. Then they were either selected for CD4^+^ T cells (CD3^+^CD4^+^) or CD8^+^ T cells (CD3^+^CD8^+^) before analyzing cytokine production for IFN-γ, TNF-α and IL-2 using a similar gating strategy.

**Figure 2:**
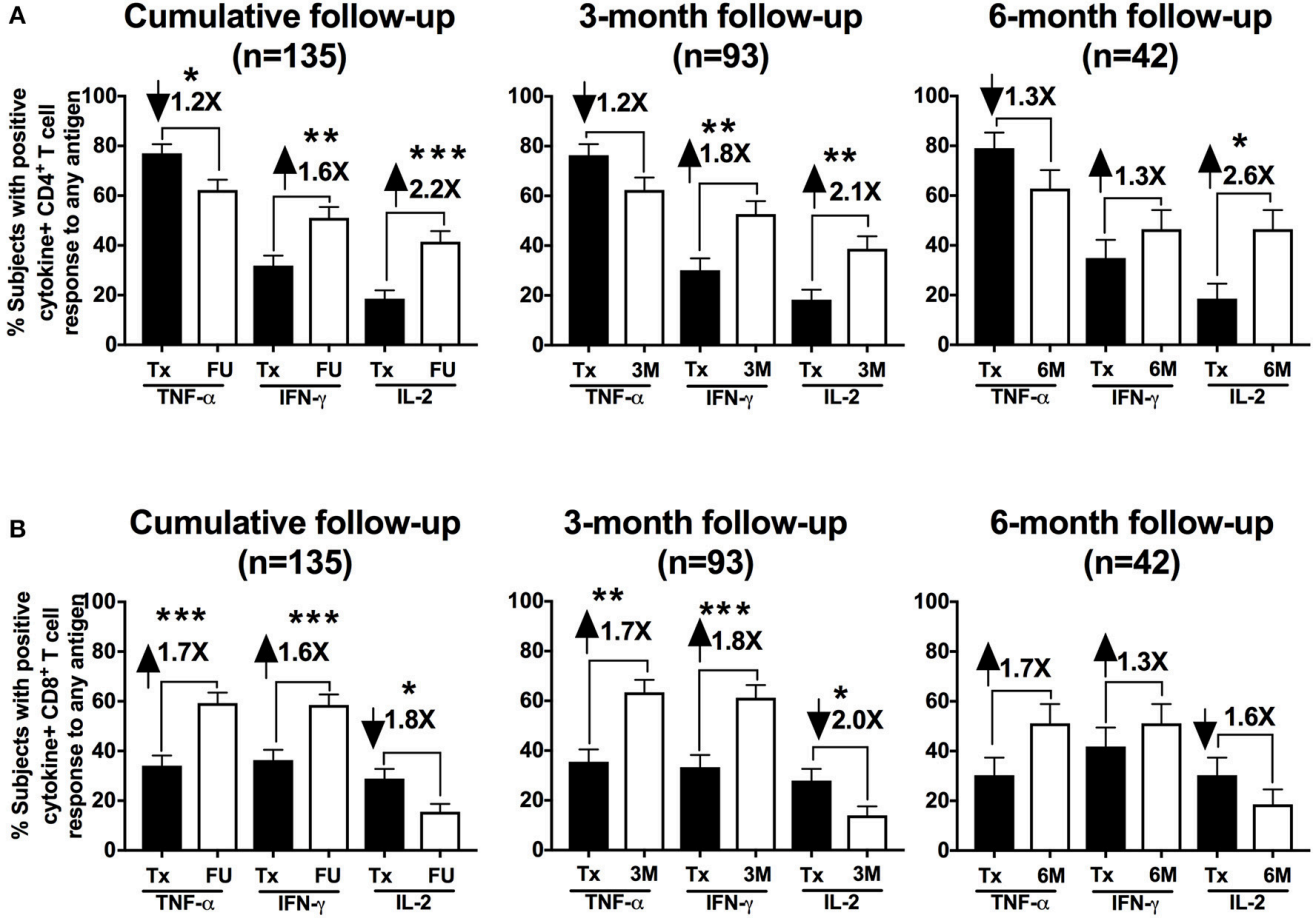
Th1 cytokine hierarchy changed from TNF-α-producing to IFN-γ-producing CD4^+^ T cells in chlamydia-infected women after therapy: Bar graph representing the change in the percentage of subjects with positive CD4^+^ **(A)** and CD8^+^ **(B)** cytokine-positive T cell responses from the Tx (black bars) to FU visits (white bars) among a cumulative 135 women and further stratified in 93 women at a 3-month visit and 42 women at a 6-month visit. Standard error of mean (SEM) is shown on the bars. The numbers on top of the bars represent the fold change in the percent positive CD4^+^/CD8^+^ cytokine positive responses from Tx to FU visit for the respective cytokines after PBMC stimulation. An extension of McNemar's chi-square test was used to determine statistical significance. *P* < 0.05 was considered statistically significant. ***P < 0.001, **P < 0.005, and *P < 0.05.

We evaluated the frequency of CT-specific CD4^+^ and CD8^+^ TNF-α, IFN-γ, and/or IL-2 responses in the stimulated PBMCs using intracellular cytokine staining. After PBMCs were stimulated with CT antigens (CT EBs, recombinant Pgp3, and MOMP peptides), T cells were gated from a singlet population of lymphocytes gate by selecting either the CD3^+^CD4^+^ population (CD4^+^ T cells) or CD3^+^CD8^+^ population (CD8^+^ T cells), and were subsequently selected for IFN-γ- and/or TNF-α-, and/or IL-2- producing populations for both CD4^+^ and CD8^+^T cells (Figure 1). In the 135 participants for whom CT-specific CD4^+^ and CD8^+^ TNF-α, IFN-γ, and IL-2 responses were evaluated at follow-up (93 3-month visits and 42 6-month visits), we found a significant decline in the frequency of a positive CD4^+^TNF-α response from the treatment visit to the follow-up visit (77 vs. 62%, *P* = 0.029, Figure 2A, left panel). Stratifying these responses into 3-month and 6-month visits yielded similar trends (Figure 2A middle and right panels), though they did not achieve significance (possibly due to smaller sample size). In contrast, we saw a significant increase in the frequency of positive CD4^+^IFN-γ (from 31.8 to 51.1%, *P* = 0.002) and CD4^+^IL-2 (from 18.5 to 41.5%, *P* = 0.001) responses from the treatment visit to follow-up (cumulative), respectively (Figure 2A left panel). Similar trends for CD4^+^IFN-γ responses were seen for both 3-month (*P* = 0.002) and 6-month visits (*P* = 0.352; Figure 2A middle and right panel). We also observed a significant increase in the frequency of positive CD8^+^TNF-α (from 34.1% to 59.3, *P* = 0.001) and CD8^+^IFN-γ (from 36.3 to 58.5%, *P* = 0.001) responses from the treatment visit to follow-up, while a notable decline was found in frequency of CD8^+^IL-2 responses from the treatment visit to follow-up as seen cumulatively (*P* = 0.024; Figure 2B left panel) and for 3-month (*P* = 0.017) (Figure 2B middle panel) and 6-month (*P* = 0.385) visits (Figure 2B right panel), respectively.

**Figure 3:**
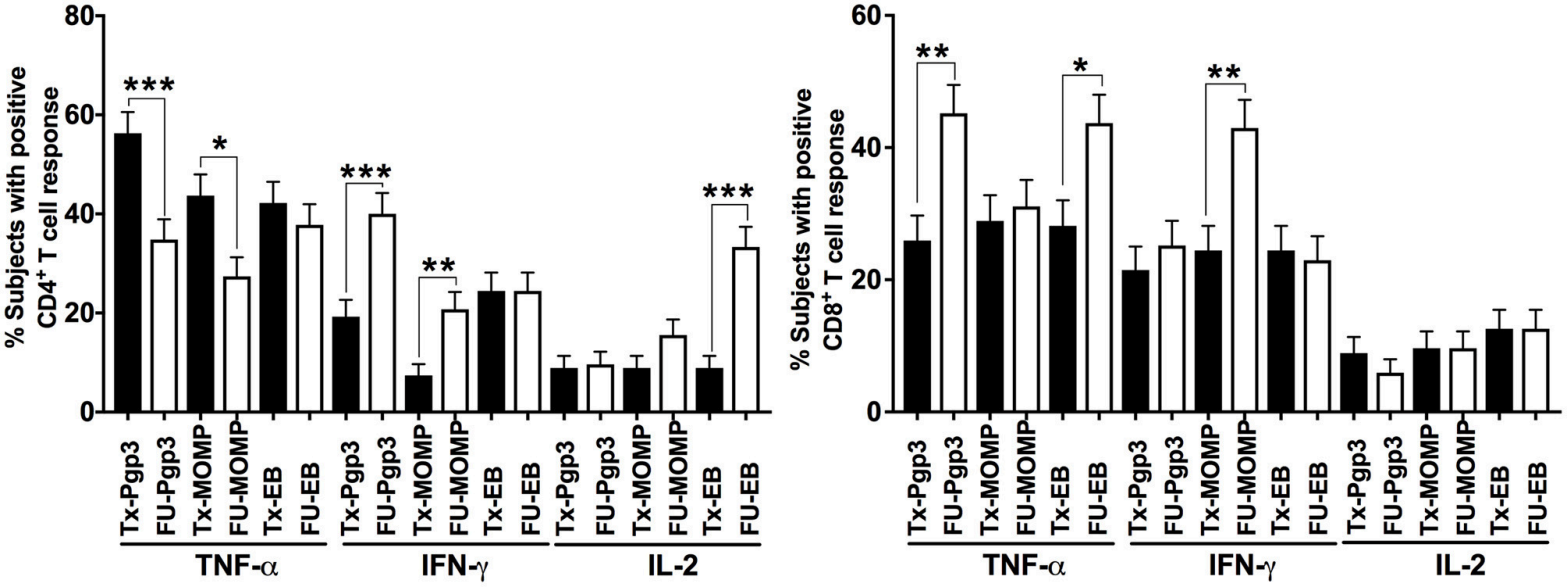
T cell responses against individual *Chlamydia trachomatis* antigens: Bar graph representing the change in the percentage of subjects with positive CD4^+^ **(Left)** and CD8^+^ **(Right)** cytokine-positive T cell responses from the Tx (black bars) to FU visits (white bars) among 135 women (cumulative). Standard error of mean (SEM) is shown on the bars. An extension of McNemar's chi-square test was used to determine statistical significance. *P* < 0.05 was considered statistically significant. ***P < 0.001, **P < 0.005, and *P < 0.05.

Next, we evaluated whether these changes in the differential cytokine responses between visits were associated with one specific CT antigen or more than one of the CT antigens. At the treatment visit, a CD4^+^TNF-α response to Pgp3, MOMP, and EB, was detected in 56, 44, and 42%, respectively (Figure 3). A similar trend in the antigen-specific CD4^+^TNF-α response was seen at follow-up (cumulative), though the frequency of these responses was much lower (35% for Pgp3, 27% for MOMP and 38% for EB). The CD4^+^IFN-γ response at follow-up was mainly to Pgp3 (40%) and EB (24.3%). A CD4^+^IL-2 response occurred most often to EBs (33.3% at follow-up), followed by MOMP (15.6% at follow-up, Figure 3). CD8^+^TNF-α responses occurred mainly to Pgp3 (45.2%) and EB (43.7%) at follow-up, whereas the CD8^+^IFN-γ response at follow-up was primarily against MOMP (occurring in 43% of women; Figure 3). The variability in the frequency of cytokine responses to different CT antigens highlights the heterogeneity of the CT antigens contributing to the adaptive immune response against CT.

### A significant increase in the magnitude of CD4^+^ and CD8^+^ IFN-γ and TNF-α responses at follow-up was observed

**Figure 4:**
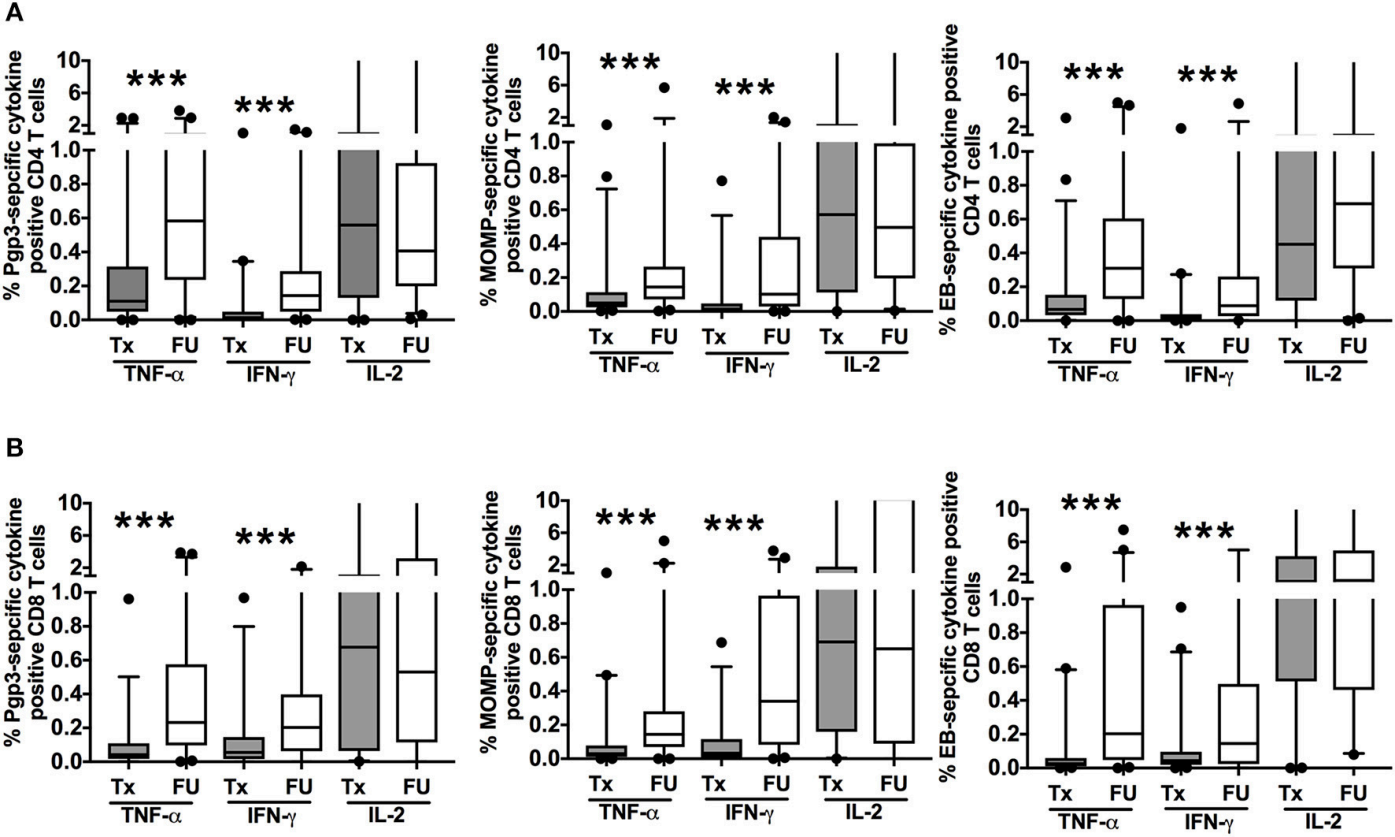
Increased magnitude of *Chlamydia trachomatis* (CT)-specific T cell cytokine responses at follow-up: Box and whisker plot with 5–95% interquartile range showing the frequency of CT-specific positive CD4^+^ T cell **(A)** and CD8^+^ T cell **(B)** responses as measured by cytokine-producing T cells after 7 h of PBMC stimulation with either recombinant Pgp3 protein (left panels), MOMP peptides (middle panels), or EB (right panels) and subtraction of the background responses at treatment (Tx, gray boxes) or follow up (FU, open boxes) visits in 135 women with CT infection at the Tx visit. Compared to the Tx visit, women had a stronger magnitude of CD4^+^ and CD8^+^ TNF-α and IFN-γ T cell responses detected at FU (*P* < 0.001). Line within the box represents the median and the symbols above and below the whiskers represent the outliers that are either greater than 95th or less than 5th percentile. A log transformed mixed model was used to compare Tx vs. FU. *P* < 0.05 was considered statistically significant. ***P < 0.001.

To evaluate the change in magnitude of T cell responses at the treatment visit and at follow-up, we measured the proportion of cytokine-producing CT-specific T cells in PBMCs from treatment and follow-up visits (cumulative) upon stimulation with the same 3 CT antigens. Although there was a decline in the frequency of a positive CD4^+^TNF-α response at follow-up, we observed a significant increase in the magnitude of the CD4^+^TNF-α response from treatment to follow-up in participants who had a positive response at both visits (*P* < 0.001 for Pgp3, MOMP, and EB, Figure 4A). Similarly, there was an increase in the magnitude of the CD8^+^TNF-α response at follow-up (*P* < 0.001 for Pgp3, MOMP, and EB, Figure 4B). We also observed a significant increase in the magnitude of CD4^+^IFN-γ and CD8^+^ IFN-γ responses at follow-up (*P* < 0.001 for Pgp3, MOMP, and EB for both CD4^+^ and CD8^+^ responses), while the magnitude of the IL-2 response remained mostly unchanged between visits (Figures 4A, B). Overall, there was a stronger magnitude of IFN-γ- and TNF-α-producing T cell responses at follow-up after therapy.

### A CD4^+^IFN-γ response at follow-up was detected more often in women without reinfection compared to those with reinfection

**Figure 5:**
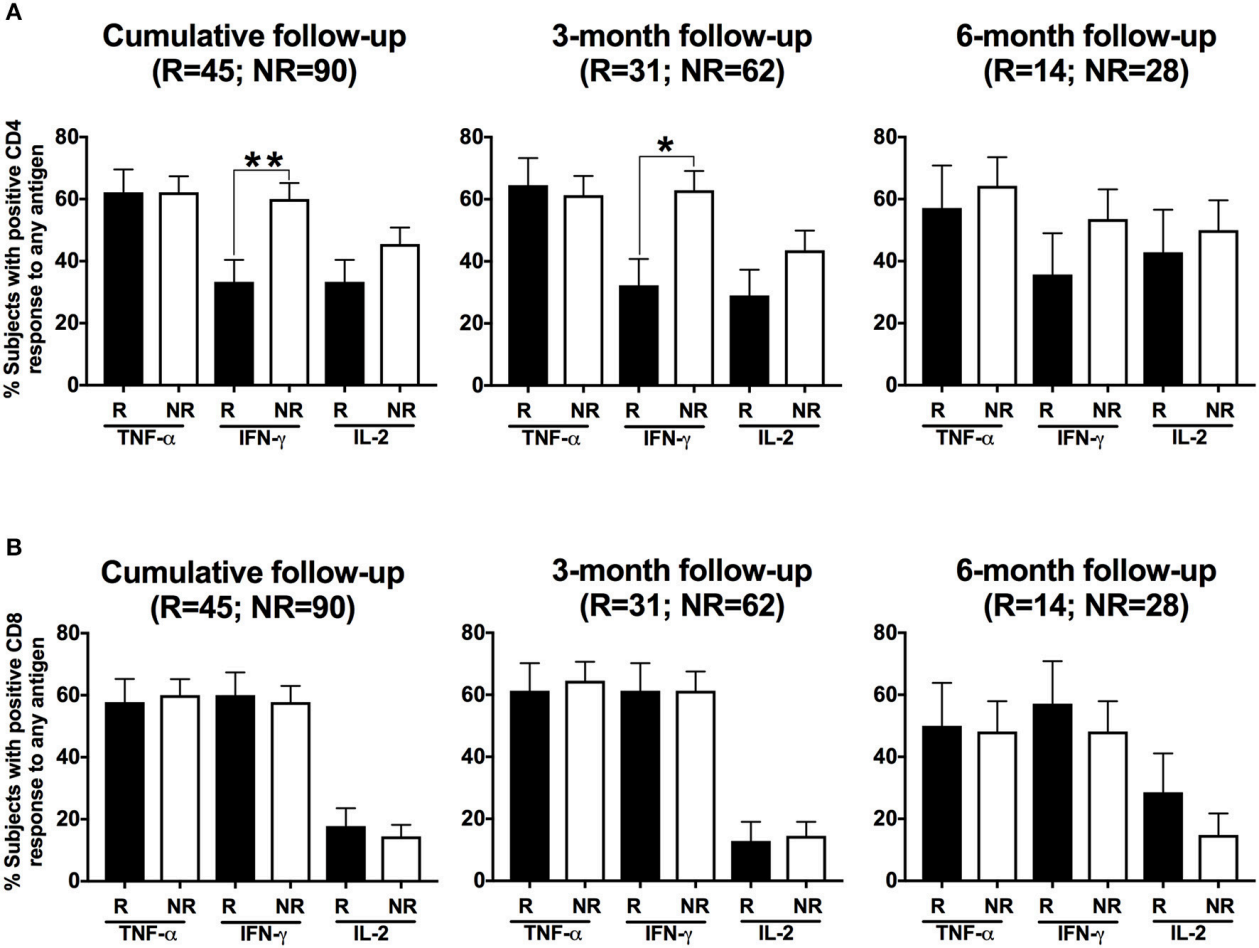
Women with a CD4^+^IFN-γ response detected at follow-up had a lower frequency of *Chlamydia trachomatis* (CT) reinfection: Bar graph illustrating the percentage of women with positive CD4^+^ T cell **(A)** and CD8^+^ T cell **(B)** responses detected after their PBMCs were stimulated with any of the three CT antigens (Pgp3, MOMP, EB), with women stratified based on their CT status at follow-up (R = reinfection, NR = no reinfection). A significantly higher proportion of women from the NR group (white bars) had a CD4^+^ IFN-γ response detected than women from the R group (black bar; *P* = 0.005; OR 3.00 [95% CI 1.40, 6.42]) for cumulative follow-up. Standard error of mean (SEM) is shown on the bars. An extension of McNemar's chi-square test was used to compare R vs. NR matched groups. *P* < 0.05 was considered statistically significant. **P < 0.005, and *P < 0.05.

To determine whether a specific adaptive T cell cytokine response was associated with protection against CT reinfection, we compared detection of T cell cytokine responses at follow-up in women with vs. without CT reinfection. We first analyzed the percentage of participants whose stimulated PBMCs mounted CD4^+^ and CD8^+^ cytokine responses (IFN-γ, TNF-α and IL-2) to any of the CT antigens at follow-up and the relationship to reinfection status. A CD4^+^IFN-γ response was detected more often in women without reinfection compared to those with reinfection when analyzed cumulatively (60 vs. 33%, *P* = 0.005; OR = 3.00 [95% CI: 1.40, 6.42]; Figures 5A left panel). Upon stratifying these responses for 3-month and 6-months follow-up visits, we found that the association remained significant at the 3-month visit (*P* = 0.008), but not the 6-month visit (*P* = 0.289). There was no significant association of other CD4^+^ cytokine responses with reinfection status (Figure 5A), nor was there an association of a CD8^+^ cytokine response with reinfection status (Figure 5B). Since women from the reinfected group more often reported unprotected sex after treatment than women from the group without reinfection (*P* = 0.005), we performed a multivariate analysis (on cumulative data) examining the relationship between CD4^+^IFN-γ response and reinfection status that controlled for unprotected sex. Our analysis revealed that women with a positive CD4^+^IFN-γ response had significantly lower odds of reinfection (*P* = 0.002; OR = 0.28; [95% CI: 0.12, 0.62]) than those with a negative CD4^+^IFN-γ response after controlling for unprotected sex in the analysis. In a subanalysis, we also analyzed whether a CD4^+^IFN-γ response at the initial treatment visit was associated with subsequent reinfection at follow-up and found no association of a CD4^+^IFN-γ response at the treatment visit with subsequent reinfection (*P* = 0.603) either cumulatively or for both the follow-up visits separately. This suggests that the timing of the CD4^+^IFNγ response is an important factor in providing protective immunity against CT reinfection.

**Figure 6:**
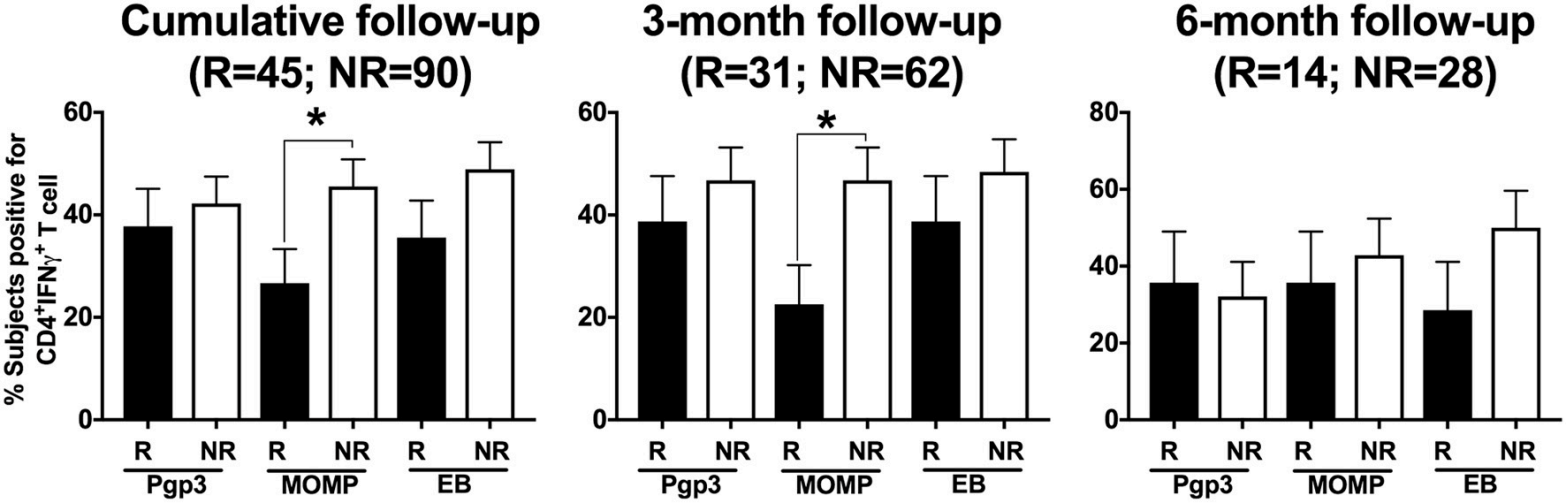
Differential and temporal CD4^+^IFN-γ T cell response with different *Chlamydia trachomatis* (CT) antigens. Bar graph depicting the percentage of women with a CD4^+^IFN-γ T cell response detected after their PBMCs were stimulated with any of the three CT antigens (Pgp3, MOMP, EB), with women stratified based on their CT status at follow-up (R = reinfection, NR = no reinfection). Stimulation with different CT antigens led to differential CD4^+^IFN-γ responses, with MOMP stimulation resulting in significantly higher CD4^+^IFN-γ T cell response from women in the NR group compared to women in the R group at 3-month and 6-month follow-up visit respectively. Standard error of mean (SEM) is shown on the bars. An extension of McNemar's chi-square test was used to compare R vs. NR matched groups. *P* < 0.05 was considered statistically significant. *P < 0.05.

In another subanalysis investigating the frequency of a CD4^+^IFN-γ response at follow-up to each of the 3 CT antigens in both participant groups, we found that the significant association of a CD4^+^IFN-γ response in women without CT reinfection was driven primarily by CD4^+^IFN-γ responses to MOMP (*P* = 0.039) when analyzed cumulatively (Figure 6). Upon stratification by follow-up visit, the association remained significant for the 3-month follow-up visit (*P* = 0.031), but not the 6-month follow-up visit (*P* = 0.661).

**Figure 7:**
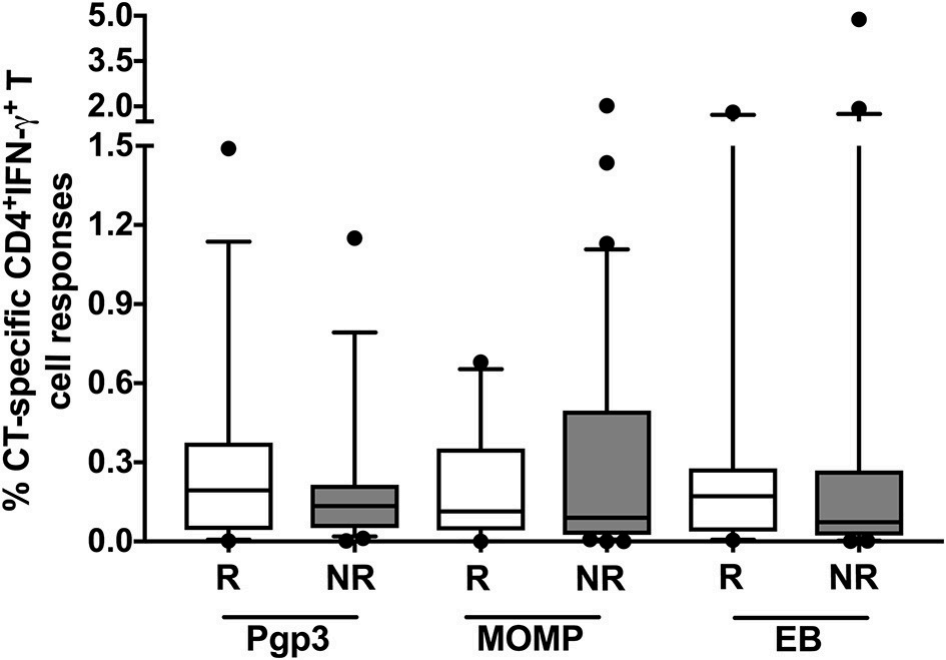
No difference in the magnitude of the CD4^+^ IFN-γ response detected in women with vs. without reinfection at follow-up: Box and whisker plot with 5–95% interquartile range showing the percentage of *Chlamydia trachomatis* (CT)-specific IFN-γ-producing CD4^+^ T cells at follow-up when stimulated with CT antigens: Pgp3, MOMP and EB. No significant difference in the magnitude of the CD4^+^ IFN-γ response in the reinfection (R) group (*n* = 45; white box) vs. the no reinfection (NR) groups (*n* = 90; gray box). Line within the box represents the median and the symbols above and below the whiskers represent the outliers that are either greater than 95th or less than 5th percentile. A log transformed mixed model was used to compare R vs. NR matched groups. *P* < 0.05 was considered statistically significant.

To investigate whether the magnitude of the CD4^+^IFN-γ response differed at the follow-up visit in women who did vs. did not have reinfection, we evaluated the proportion of cytokine-producing CT-specific T cells in both groups at follow-up. Even though we had found a cumulative difference in the frequency of a CD4^+^IFN-γ response at follow-up between those who did vs. did not have reinfection, we did not observe any significant difference in the magnitude of the CD4^+^ T cell IFN-γ responses between the groups at follow-up (Figure 7). We also did not observe any significant difference in the mean fluorescent intensity of CD4^+^IFN-γ (data not shown) at follow-up time points, suggesting these cells also do not differ significantly in terms of amount of IFN-γ production per cell basis. This observation in conjunction with the above-mentioned results suggest that the timing of a CD4^+^IFN-γ response may be more important than the magnitude of the response itself in providing protection against CT reinfection.

### In women with a CT-specific CD4^+^IFN-γ response detected at follow-up, the CD4^+^IFN-γ-producing T cells predominantly co-produced either TNF-α or IL-2

**Figure 8:**
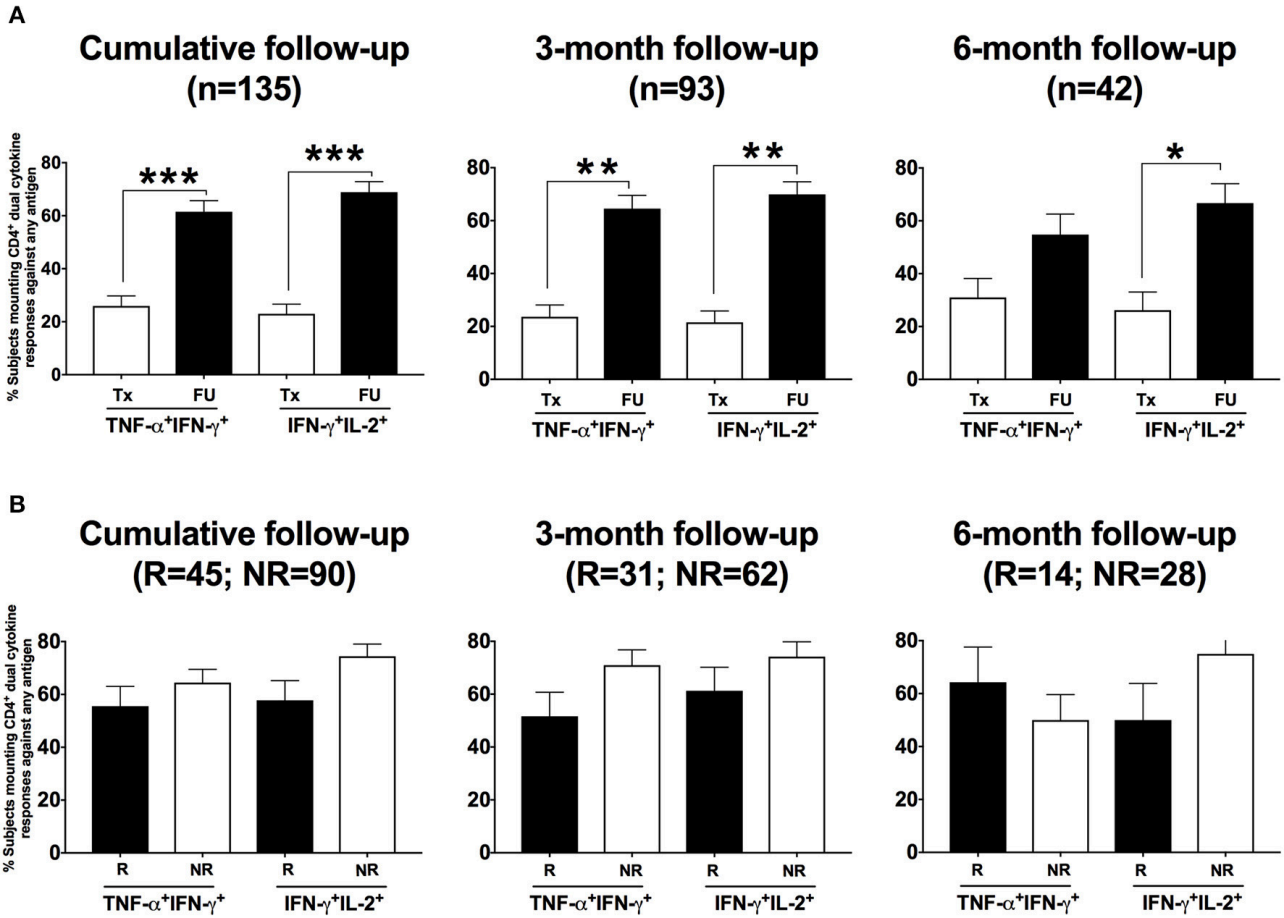
Women presenting for follow-up after chlamydia treatment predominantly had a dual cytokine-producing CD4^+^ T cell response detected: Percentage of women with CD4^+^ dual TNF-α IFN-γ responses and IFN-γ IL-2 responses detected after their PBMCs were stimulated with any of the three *Chlamydia trachomatis* (CT) antigens among those with a positive CD4^+^ IFN-γ response. **(A)** Bar graph showing the difference in the percentage of women with CT infection at enrollment who had a CD4^+^ dual cytokine response detected between the two visits: treatment (Tx) visit (white bars) and follow-up (FU) visit (black bars). A significantly higher proportion of women at the FU visit had a CD4^+^ dual cytokine response detected compared to those at the Tx visit (*P* < 0.001). **(B)** Bar graph depicting the difference in the percentage of women with CD4^+^ dual TNF-α +FN-γ responses and IFN-γ+IL-2 responses detected (determined using dual gating) from the reinfection (R) (white bars) vs. the no reinfection (NR) (black bars) groups, after their PBMCs were stimulated with any of the three CT antigens. There was no significant difference in the frequency of these dual CD4^+^ cytokine responses between women from the R and NR groups. Standard error of mean (SEM) is shown on the bars. An extension of McNemar's chi-square test was used to compare R vs. NR matched groups. *P* < 0.05 was considered statistically significant. ***P < 0.001, **P < 0.005, and *P < 0.05.

As dual cytokine-producing T cells have been shown in general to be functionally superior over single cytokine-producing cells (23) and correlated with protection against chlamydia in murine models (24), we examined the CD4^+^ T cells from women who had positive CD4^+^IFN-γ responses for their ability to co-produce TNF-α or IL-2. Using dual gating, we determined the proportion of women with CD4^+^IFN-γ-producing T cells that were dual positive either for TNF-α or for IL-2 for both treatment and follow-up visits. At the follow-up (cumulative) compared to the treatment visit, we found a significantly higher percentage of women had CD4^+^ T cells that co-produced IFN-γ with TNF-α (61 to 26%, *P* < 0.001) and IFN-γ with IL-2 (69 to 23%, *P* < 0.001; Figure 8A). The association remained significant for the 3-month visit (*P* < 0.001 for both IFN-γ +TNF-α+ and IFN-γ +IL-2+) and still trended for the 6-month visit (*P* = 0.067 for IFN-γ +TNF-α+ and *P* = 0.014 for IFN-γ +IL-2+). Upon evaluating the relationship of these dual cytokine responses at follow-up with reinfection status, we did not find any significant association (Figure 8B). This suggests that although CT-specific CD4^+^ T cells that co-produce IFN-γ with either TNF-α or IL-2 are a prominent component of the adaptive immune response to CT, it is the CD4^+^ IFN-γ response that is essential for protective immunity to CT reinfection.

## Discussion

Lack of knowledge of the immune correlates of protection against CT in humans has hampered efforts in developing a vaccine against this highly prevalent STI that causes substantial morbidity in women. The murine model of CT infection clearly supports a Th1 IFN-γ response as necessary for eradication of primary CT infection and a correlate for protective immunity against CT reinfection upon re-exposure (12, 13, 25). However, the limited human research to date on CT-specific adaptive immune responses had produced contradictory results with respect to whether Th1- vs. Th2-associated cytokine responses predominated in human CT infection (16, 17, 26, 27), and the studies were limited by smaller samples sizes.

Our current study builds on our earlier study in which we used ICS to demonstrate that the majority of CT-infected women presenting for treatment of their CT infection had a positive CD4^+^TNF-α response after stimulation of their PBMCs with CT antigens (18). In this follow-up study, we investigated their CT-specific CD4^+^ and CD8^+^ IFN-γ, TNF-α, and IL-2 responses at follow-up after treatment and evaluated for association of a specific cytokine response with lower risk for CT reinfection. There was a significant increase in the frequency of CD4^+^ and CD8^+^ IFN-γ responses from treatment to follow-up and subsequently, there was a significant decline in the frequency of CD4^+^TNF-α responses. These results suggest that the early response upon exposure to CT in humans may be a CD4^+^TNF-α response, however as the adaptive immune response evolves, it appears to be strongly driven by IFN-γ-producing CD4^+^ T cells that often co-produce other Th1 cytokines. It is possible that this transition reflects the inability of CD4^+^TNF-α-producing T cells to eradicate the infection alone, which seems to be supported by the finding that the majority of TNF-α-producing CD4^+^ T cells before treatment did not co-produce IFN-γ or IL-2, whereas at follow-up, the majority of IFN-γ-producing CD4^+^ T cells co-produced either TNF-α or IL-2, a phenotype highly indicative of functionally superior cells (23). An effector cytokine such as IFN-γ could synergize the existing anti-CT activity of TNF-α for better protection (28). However, our finding that CD4^+^ T cells which co-produced IFN-γ with either TNF-α or IL-2 (based on dual gating) were not associated with protection against reinfection seemed to suggest that a CD4^+^IFN-γ T cell response is sufficient for immune protection against CT reinfection; this has also been described for infection from another Gram-negative bacteria, *Salmonella typhi* (29). Our results are also consistent with our previous findings that CT-antigen specific responses are quite heterogeneous (18). We found differential cytokine responses by antigen-specific T cells at follow-up, with the CD4^+^IFN-γ response mainly elicited by CT Pgp3 and EB. Our findings are relevant from a vaccine development perspective, as our results suggest that more than one CT antigen may be required as vaccine candidate for eliciting an effective immune response against CT.

It has been hypothesized that early treatment of CT infection in humans could hinder the development of protective immunity in some individuals, a concept known as “arrested immunity” (6, 9). In our study, we found that at follow-up after CT-infected women were treated, there was a higher percentage of women who had a CD4^+^IFN-γ response detected and the magnitude of the response was stronger than the frequency and magnitude of these responses at the treatment visit, irrespective of their reinfection status. This suggests that in our study population, antibiotic treatment did not completely prevent the development of some degree of adaptive immunity with a CD4^+^IFN-γ response that might aid in more rapidly clearing infection in those who have reinfection. However, we were limited in not knowing the duration of CT infection prior to treatment.

Our study provides clear evidence in humans that a CD4^+^IFN-γ response may be an immune correlate of protection against CT reinfection and adds substantial evidence to existing knowledge of the human immune responses in CT-infection. We found that women who had a CD4^+^IFN-γ response detected at follow-up were significantly less likely to have reinfection, though the protection might not be long lasting, as possibly suggested by our analyses of CD4^+^IFN-γ response stratified by 3-month vs. 6-month follow-up visit. Further analysis of the CD4^+^IFN-γ response revealed that the responses against CT-MOMP peptides were driving the association of a CD4^+^IFN-γ response with protection against reinfection, implicating an existence of differential immune dominance among women with and without CT reinfection. Our analysis also revealed that among those with a CD4^+^IFN-γ response detected, there was no association between the magnitude of the CD4^+^IFN-γ response and reinfection status, suggesting it is not the magnitude of the CD4^+^IFN-γ response, rather the timing of the response that influences reinfection risk. From a clinical perspective, while a CD4^+^IFN-γ response appears to provide protection against CT reinfection, we were unable to determine whether some individuals had complete or partial immunity, the latter which would likely manifest as a reinfection that is more short-lived due to more rapid and effective immune-mediated clearance. Additional insights into the degree of protective immunity would require a study with more frequent CT testing.

One of the limitations of our study was that our study population was primarily comprised of African-American women, and it is unclear if our findings can be extrapolated to women of other race/ethnicities or to men. Our study focused on cytokines associated with Th1 responses (against 3 CT antigens) due to the animal model data highlighting the essential role of Th1 in protective immunity (13, 14, 30–32); however, we did not investigate the contribution of Th2 responses in CT immunity, which may be important since a previous publication demonstrated that robust Th2 responses have been detected in some CT-infected women (26). We did not have mucosal mononuclear cells (MNCs) for evaluating these same immune responses by ICS, so it is unknown if our findings based on PBMC responses will translate to the immune responses seen at the mucosal site. It is possible that at the treatment visit, there were a significant proportion of IFN-γ-producing CD4^+^ T cells residing in the mucosa and thus were not measured in our PBMC studies. We realize that the immune responses against CT could be not only temporal in nature, but could also vary between the peripheral blood and the mucosa, as the gene expression of CT is developmentally regulated and thus different immune responses could be directed against different proteins expressed. Thus, the same antigens may or may not mount similar responses at the mucosa as seen in periphery, and there could be different immune dominant epitopes. Although a higher proportion of women in the reinfection group reported having unprotected sex between the treatment and follow-up visits, the immunological relevance of this finding is not known. It could be possible that some women who had more unprotected sex were more frequently exposed to CT in a short period, which could influence the type or magnitude of their immune responses; however, with CT testing limited to the follow-up visit, our study was not designed to identify frequency of CT exposures. While we also found a significant increased magnitude of the IFN-γ-producing CD4^+^ T cells at the follow-up visit, without memory markers we cannot determine if they are expanded memory cells (effector memory) left after primary infection (which is highly likely) or they were cells recruited back from the mucosa after primary infection is cleared (central memory). While we found a trend toward lower reinfection frequency in those with prior chlamydia, our prior chlamydia variable was limited in that it was based on participant self-report and medical record review of chlamydia test results from only the study clinic. Therefore, there may have been some misclassification of prior chlamydia status, which was most likely an underestimate of the proportion of subjects with prior CT infection.

In future studies, we plan to decipher the role of Th1 responses at the primary site of infection (mucosal site) and to validate that CT-specific IFN-γ-producing CD4^+^ T cells traffic to mucosa and are directly involved in providing protection. We will also evaluate the immune dominance of various CT-antigens to identify specific epitopes that may be the primary target of the immune mediated response against CT. We will also determine whether the IFN-γ-producing CD4^+^ T cells at follow-up are of memory origin; in general, memory T cells mount a stronger and more robust T cell response upon re-challenge with a pathogen (33). We also plan to investigate if one memory subset is dominant over other in women without reinfection vs. with reinfection.

In conclusion, women with CT infection had an adaptive immune response to CT detected after therapy that was more CD4^+^IFN-γ centric compared to the TNF-α dominant response prior to treatment. A CD4^+^IFN-γ response was more often detected at follow-up in women without reinfection vs. those with reinfection, suggesting CD4^+^IFN-γ may provide some degree of protective immunity against CT reinfection and could serve as a correlate of protection, which would be important in future research studies evaluating CT antigens for CT vaccine development or evaluating CT screening strategies based on immune correlates.

## Author contributions

CP and WG conducted clinical procedures. RB, KG, SJ, and WG designed the laboratory experiments. RB conducted the laboratory experiments. XC and SL performed statistical analyses. RB, KG, SJ, CP, WG, XC, and SL interpreted the analyses and contributed to writing the manuscript.

### Conflict of interest statement

The authors declare that the research was conducted in the absence of any commercial or financial relationships that could be construed as a potential conflict of interest.
